# Developing biomedical engineering technologies for reproductive medicine

**DOI:** 10.1002/SMMD.20220006

**Published:** 2022-12-27

**Authors:** Yujuan Zhu, Bin Kong, Rui Liu, Yuanjin Zhao

**Affiliations:** ^1^ Department of Rheumatology and Immunology Nanjing Drum Tower Hospital School of Biological Science and Medical Engineering Southeast University Nanjing China; ^2^ Oujiang Laboratory (Zhejiang Lab for Regenerative Medicine, Vision and Brain Health) Wenzhou Institute University of Chinese Academy of Sciences Wenzhou Zhejiang China

**Keywords:** 3D printing, biomaterials, biomedical engineering, infertility, microfluidic technology, reproductive medicine

## Abstract

Infertility is a rising global health issue with a far‐reaching impact on the socioeconomic livelihoods. As there are highly complex causes of male and female infertility, it is highly desired to promote and maintain reproductive health by the integration of advanced technologies. Biomedical engineering, a mature technology applied in the fields of biology and health care, has emerged as a powerful tool in the diagnosis and treatment of infertility. Nowadays, various promising biomedical engineering approaches are under investigation to address human infertility. Biomedical engineering approaches can not only improve our fundamental understanding of sperm and follicle development in bioengineered devices combined with microfabrication, biomaterials, and relevant cells, but also be applied to repair uterine, ovary, and cervicovaginal tissues and restore tissue function. Here, we introduce the infertility in male and female and provide a comprehensive summary of the various promising biomedical engineering technologies and their applications in reproductive medicine. Also, the challenges and prospects of biomedical engineering technologies for clinical transformation are discussed. We believe that this review will promote communications between engineers, biologists, and clinicians and potentially contribute to the clinical transformation of these innovative research works in the immediate future.

## Graphical abstract

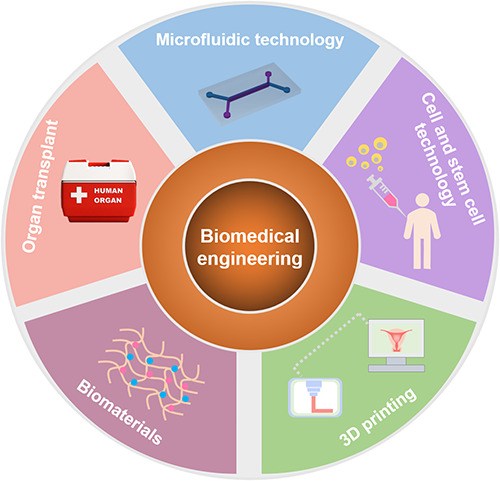

1

**Key points**

- A comprehensive summary of biomedical engineering technologies and their applications in reproductive medicine.
- The challenges and prospects of biomedical engineering technologies for clinical transformation.

## 2 INTRODUCTION

**FIGURE 1:**
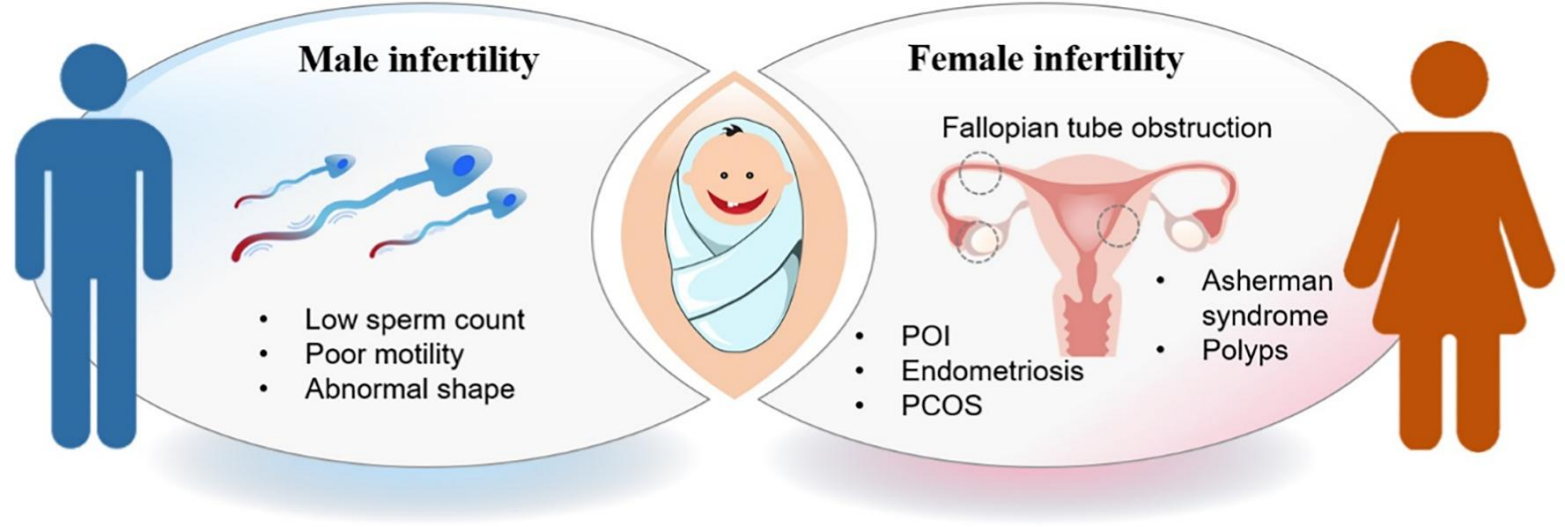
Schematic illustration of male and female infertility. POI, primary ovarian insufficiency; PCOS, polycystic ovary syndrome.

Infertility is considered as a complicated disorder with far‐reaching biological, psychosocial, and economic implications.^1^, ^2^ It refers to the failure to conceive over a year of regular unprotected intercourse, and about 1 in every 10 couples worldwide are infertile at reproductive age.^3^, ^4^, ^5^ Successful pregnancy is a coordinated process that involves the elegant interplay between distinct physiological events occurring in both the male and female. In order to get pregnant, minimum requirements are required including ovulation, mating‐competent gametes, sperm–oocyte interaction and fertilization in the reproductive tract, embryo transport, and implantation in the uterus.^6^ Therefore, a series of complex risk factors can lead to infertility, mainly including the abnormalities in the production of the competent oocyte and sperm, the embryo implantation, as well as the defects in sperm transport through the female reproductive tract (Figure 1).^7^ The development of techniques for infertility treatment could be traced back to the period when spermatozoa was discovered in 1677.^8^, ^9^, ^10^ By then, advancements were made in medical science to understand the reproductive physiology, process of gamete interaction, and infertility treatment, especially in the past three decades.

Biomedical engineering, also referred to as bioengineering, is a multidisciplinary field that merges engineering and medicine, applying engineering principles and design concepts to advance technology and improve healthcare. This field intends to bridge the gap between engineering and medicine to advance healthcare treatment. As such, biomedical engineering has been at the forefront of many medical advances spanning a broad array of subfields in recent years. Many of the biomedical engineering approaches have been successfully applied for advanced prosthetics,^11^ artificial organs,^12^ surgical robots,^13^ pharmaceutical drugs,^14^ medical therapy,^15^ and so on. As one of the most visible contributions of biomedical engineering, various therapeutic devices have been developed, such as cochlear implant and vascular stent technology. Also, tissue engineering has emerged as clinical realities for tissue repair, including cartilage, bone, liver, kidney, blood vessels, and skeletal muscle. These progresses have demonstrated that biomedical engineering is highly attractive to improve the quality of people's lives with advanced methodologies. To date, engineering technologies together with distinct features have been extensively developed in reproductive health, displaying the enormous potential to addressing the limitations of infertility treatment with traditional techniques.

In this review, we first give a snapshot of male and female infertility and then summarize the recent advances of biomedical engineering and its application in the field of reproductive medicine, from in vitro cell models to clinical therapies with the ultimate goal of achieving fertilization. Many technologies have been integrated in this interdisciplinary field, including microfluidics, organ transplant, biomaterials, cell and stem cell, as well as three‐dimensional (3D) printing. Here, we will summarize the recent state‐of the‐art approaches that are utilized in human reproductive diseases and comprehensively review recent research findings and clinical advances in reproductive medicine. Also, the challenges and prospects of biomedical engineering technologies for clinical transformation are discussed.

## 3 SNAPSHOT OF INFERTILITY

### 3.1 Male infertility

Male infertility refers to the inability of a male to impregnate a fertile female, and it accounts for approximately 20% infertility in humans.^16^, ^17^ A downward trend in male fertility highlights the need for accurate diagnosis and effective treatment. Currently, many studies have reported that a wide variety of risk factors could reversibly or irreversibly influence sperm quality, such as alcohol, drugs, overweight, mental diseases, infection, and prolonged exposure to various environmental factors including industrial chemicals, radiation, and overheating.^18^ According to clinical observations, male infertility is commonly caused by deficiencies in the semen, because semen quality is vital to maintain healthy fertilizing ability of sperm. As a surrogate measure for male fecundity, semen quality is commonly evaluated in the diagnosis of male infertility, which is essentially carried out at high standards in the collection and measurement of male ejaculate. It is performed to analyze certain characteristics of sperms, including the number, shape, motility, vitality, and production.

The primary function of the sperm is to reach the ovum and fuse with it to deliver genetic materials; thereby, the motility, capacitation, acrosome reactivity, and ultimately, fertilization of the oocyte are key events regarding successful pregnancy.^19^, ^20^ To understand the main determinants of sperm function, we must first consider the fundamental structure of the sperm cell. In humans, a human sperm consists of two distinguishable parts, a flattened pear head and a slender tail. The sperm head is featured with a minimum of cytoplasm and a compact nucleus and functions to deliver a haploid set of chromosomes to the oocyte. The sperm tail propels the sperm at the rate of 1–3 mm/min by whipping in an elliptical cone and gives the sperm an increased chance of penetrating the boundaries of the female reproductive tract to reach the egg.

#### 3.1.1 Immune infertility

Immune infertility is termed as the reproductive failure due to the impaired sperm–oocyte interactions in the female reproductive tract. In these cases, antisperm antibodies (ASAs) are observed to be far more frequent than anti‐oocyte antibodies. ASA is known to be responsible for up to 30% of infertile couples, and the first type of ASA was described in animals in 1954 by Rumke with the cytotoxic, immobilizing, and agglutinating functions.^21^, ^22^ Sperm is considered as being antigenic toward the female body; thus, ASAs are naturally produced by the immune system to attack and eliminate sperm.^23^ Consequently, a series of sperm activities were interfered, including the poor sperm motility and transport through the female reproductive tract, inhibited capacitation and acrosome reaction, defective fertilization, and embryonic development. There are many risk factors for ASA in men, such as inflammation of the male reproductive tract (MRT), the breakdown of the blood‐testis barrier, genital trauma, and testicular tumors.

#### 3.1.2 Genetics

There is increasing public acceptance of the role of genetics in the causation of male infertility with the increasing use of assisted reproduction technology (ART). The genetic landscape of male infertility is highly complex, and genetic disorders could possibly explain 15%–20% of infertile men. In clinical practice, genetic disorders,^24^ including chromosomal or single‐gene disorders,^25^ mitochondrial DNA (mtDNA) mutations, chromosome aberrations, multifactorial diseases, imprinting disorders, or endocrine disorders, may lead to severe oligozoospermia and azoospermia. In recent years, with the prolonged exposure to environmental factors, epigenetic alterations in sperm have been identified to be another potential cause of male infertility.^26^ An increasing body of evidence has supported the altered epigenetic profiles during gametogenesis and germ cell maturation.

### 3.2 Female infertility

Female infertility is defined as the inability to get pregnant successfully in women of reproductive age.^27^ According to previous studies, female factors contribute to approximately 70% of the infertile cases, largely due to the highly complex process of reproduction continues in female reproductive organs. Generally, female infertility may be associated with ovaries producing eggs, the egg movement from the ovary to the uterus, the egg attachment to the uterus, and the survival of a fertilized egg or embryo after attachment. In fact, female infertility could be caused by irregular ovulation, abnormal uterine or cervix, blocked fallopian tubes, endometriosis, primary ovarian insufficiency (POI), pelvic adhesions, cancer, and cancer treatment.^28^ Other risk factors for female infertility include age, cigarette smoking, alcohol use, obesity, being underweight, as well as exercise issues.^29^ Therefore, cultivating healthy habits may increase the chances of pregnancy, while some types of infertility are not preventable.

#### 3.2.1 Ovarian disease

The ovary is one female reproductive organ in which oocytes (eggs) are developed and sex steroid hormones are released at each menstrual cycle. Therefore, ovarian reserves are a big part of pregnancy success. Notably, with approximately 30% of all infertile cases in women, polycystic ovary syndrome (PCOS) is the most common ovarian illness that affects women in their reproductive age.^30^ The telltale signs and symptoms of PCOS include cystic ovaries, ovulation irregularities, and increased androgen levels. Nowadays, mounting evidences have indicated the strong correlations of PCOS with both epigenetic and environmental traits, including diet and other lifestyle issues. In clinic, women with PCOS clearly have the increased risk for hypertension and cardiovascular disease, linked to metabolic dysfunction, in comparison to women without PCOS. Therefore, as a multifaceted syndrome, PCOS treatment is generally individualized based on the patient's presentation and desire for pregnancy in clinical practice.

POI, also known as primary ovarian failure, is a rare condition where the ovaries spontaneously stop working normally before age 40.^31^ Ovarian follicular dysfunction and ensuing deficiency in ovarian sex hormones are hallmarks of POI, thus resulting in reduced fertility, increased risks of premature mortality, osteoporosis, and cardiovascular disease, prior to the normal age of menopause. Despite that the cause of POI is unknown in most cases, the increasing evidence has displayed a wide range of factors associated with POI, including family history, age, certain diseases, and adverse life events. Currently, hormone therapy (HT) is the most common treatment,^32^ which facilitates to avoid severe symptoms and long‐term health consequences of hormone deficiency before the age of natural menopause (∼age 50).

#### 3.2.2 Tubal disease

Fallopian tubes are the delicate hollow tissue that stretch from the ovaries to the uterus in the female reproductive tract with the primary role to transport eggs. Disorders of the fallopian tube are considered among the leading causes of female factor infertility and should be specifically looked for as the site of fertilization and early embryogenesis.^33^ Tubal subfertility or infertility is credited with up to 30% of the etiology of infertility, resulting from a series of factors from congenital malformations to infections. The most prevalent cause of tubal factor infertility is a tubal disease with blockage, and the causes need be to assessed prior to treatment. In clinic, the main treatment for tubal factor infertility is usually either surgery to repair the damaged tubes or in vitro fertilization (IVF), which bypasses the fallopian tubes. Of particular note is that there are few studies evaluating the pregnancy rates between two main treatments.

#### 3.2.3 Uterine disease

The uterus is a hollow muscular organ with hormone‐responsive ability. Once the oocyte released from ovary, it could be fertilized and implanted into thickened uterine lining. Therefore, the uterus is essential for the implantation of the fertilized ovum and nourishment of the developing fetus. Recent estimates show that 1 in 500 women of reproductive age suffer from absolute uterine factor infertility.^34^ Actually, assessing for uterine factor infertility is common when a woman first presents for infertility. Intrauterine adhesions (IUAs) are described as the bands of fibrous tissues within the endometrial cavity, which is usually caused by the trauma to the basal layer of the endometrium.^35^ IUA could range from thin strings of tissue to severe cohesive adhesions (also known as the Asherman's Syndrome). Clinically, IUAs possibly lead to a series of sequelae including amenorrhea, infertility, miscarriage, and preterm birth. Endometrial hyperplasia (EH) is a pathological condition featured with the abnormal proliferation of endometrial glands and stroma, accompanied with the thickened endometrium.^36^ Of particular note is that EH is a significant concern as a clinical precursor of endometrial cancer and uterine cancer. Uterine malformation is another common group of congenital uterine anatomic abnormalities with an estimated prevalence of 6.7% in the general population.^37^ Their occurrence is associated with higher incidences of various clinical presentations ranging from reproductive disorders to life‐threatening complications although these diseases are often asymptomatic in childhood.

#### 3.2.4 Cervical disease

A considerable spread of knowledge regarding the role of the cervix in fertility has intensified over the past decades. The cervix or cervical factor is found to affect up to 5% of infertility cases. Located at the lower narrow part of the uterus, the cervix serves as a pathway allowing for the ascent of sperm for fertilization as well as preventing the entry of pathogens from the vagina into the uterus. Also, it is crucial as a protective seal during pregnancy and supports the fetus during childbirth. Clinically, infertility might be attributed to cervical mucus abnormalities, as mucus production is crucial for the transportation of sperms from the vagina to the uterine cavity. Regarding to clinical observations, cervical factor infertility occurs accompanied with a substantial reduction in sperm numbers, due to the hostile environment. Antisperm antibodies present in the cervical mucus and abnormal cervix structure could interfere with natural conception and finally lead to the infertility.^38^

## 4 RELATIVE BIOMEDICAL ENGINEERING STRATEGIES

**FIGURE 2:**
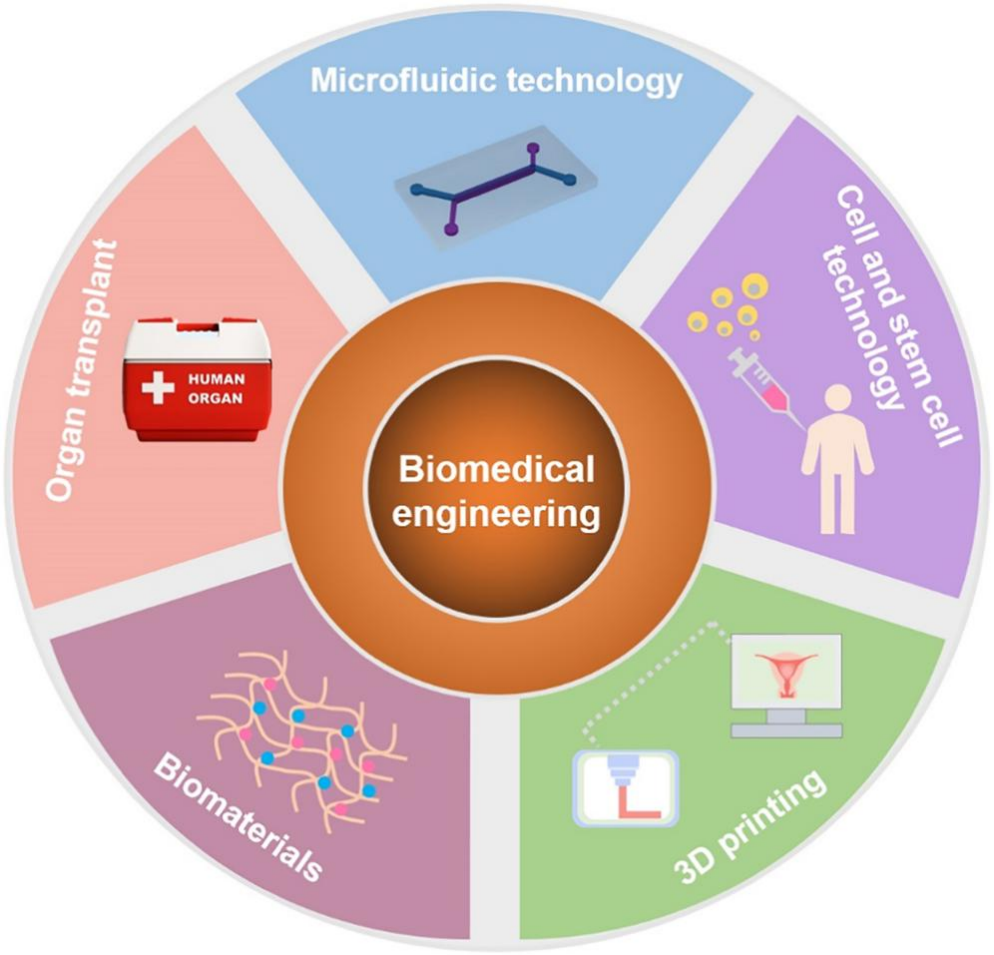
Schematic of biomedical engineering technologies relative to the treatment of infertility.

Biomedical engineering (BME) is the application of engineering theories and analytical practices to medicine and biology for diagnostic and therapeutic purposes and now has emerged as a promising field by integrating biosensors,^39^ biomaterials,^40^ artificial intelligence,^41^ and so on. To treat infertility, this field has focused heavily on infertility diagnosis, monitoring, and therapy by developing advanced materials and technologies.^42^ In the following sections, we will introduce the relative biomedical engineering techniques and discuss how these techniques advance infertility treatment and overcome shortcomings of traditional methods (Figure 2).

### 4.1 Microfluidic technology

Microfluidic technology has been first introduced in the early 1990s, which allows for the precise control and manipulation of dynamic flow at a small scale (typically sub‐millimeter) on a microfluidic chip. Microfluidic technology aims to integrate various biochemical operations onto a miniaturized chip by manufacturing microchannels and chambers.^43^ Microfluidics is considered as the science focused on the behavior of fluids through microchannels, as well as the technology related to applications in diagnosis, cell biology, single cell analysis, forensic science, and biomedical science.^44^, ^45^ Compared with traditional biological methods, microfluidics has comparative advantages for a broad range of applications due to cost‐efficiency, parallelization, ergonomics, diagnostic speed, and sensitivity.

### 4.2 3D printing

**FIGURE 3:**
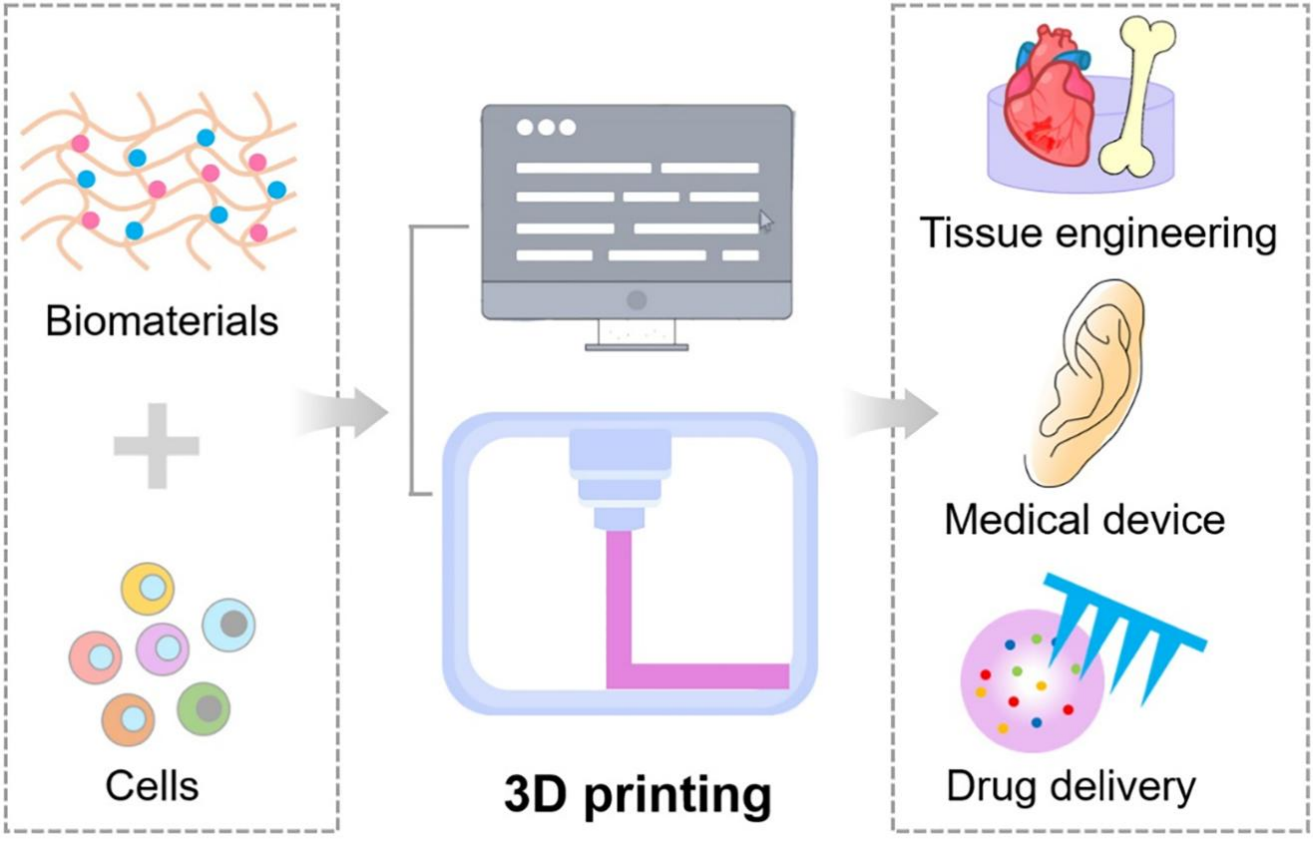
Biomedical applications of 3D printing by combination of biomaterials and cells.

3D printing has attracted an increasing interest in the last decade and widely used in the fabrication of complex structures and materials for various applications especially in the healthcare sector^46^ (Figure 3). The development of 3D printing has been largely fueled by advances in the advent of abundant cell and biomaterial sources and enormously advanced the development of biological substitutes in regenerative medicine.^47^ Generally, 3D printing is the construction of 3D objects in a highly precise and programmable manner by combining materials and other elements (such as a combination of photopolymers and cells). Nowadays, the development of 3D printing has overcome several shortcomings of traditional manufacturing techniques and provided abundant tools to create functional tissues capable of replacing diseased or damaged tissue in humans. The use of 3D printing by the manufacturing industry has a long history, and the Centre for Devices and Radiological Health at the Food and Drug Administration has reviewed and cleared 3D printed medical devices over the last decade.^48^ Moreover, advances in 3D printing have been greatly motivated by the deficiency in the supply of organs available, which are needed for current treatment of organ failure and tissue loss. As a new tool, 3D printing is highly attractive to engineer living tissues, including skin, cartilage, heart valve, and vascular grafts.^47^

### 4.3 Biomaterials

The biomaterial field is booming in recent years due to unprecedented levels of our understanding in biological systems and their interfaces with materials. Biomaterials play an integral role in medicine by engineering to interact with biological systems, where the goal is to restore tissue function and contribute to disease treatment and diagnosis.^49^ Biomaterial can be adopted from nature or synthesized in the laboratory. Generally, natural materials could be classified into proteins, polysaccharides, and decellularized scaffolds, and synthesized materials refer to materials prepared with the incorporation of metallic components, polymers, ceramics, or composite materials. To meet the needs for implantation in or on the human body, biomaterials could be flexibly designed and adapted with a range of properties to imitate key aspects of living tissues, including physiological and chemical cues and microstructure. Also, the modifications could allow biomaterials to be served as drug delivery systems,^50^ biosensors,^51^ and medical devices.^52^ As the behavior of the biomaterial largely depends on dynamic and complex environments, the deep understanding of biomaterial property and detailed cell‐material interaction is required prior to the successful performance in healthcare.

### 4.4 Cell and stem cell technology

Recent breakthroughs in transplantation of cells and stem cells have offered a powerful tool for tissue repair and regeneration.^53^, ^54^ The appropriate cell source has always been a challenge for bioengineering. To date, a wide range of cells and stem cells have been utilized for enhanced tissue repair and regeneration, such as brain, bone, heart, and pancreas islet. Generally, bioengineering uses cells to construct functional tissues beneficial for restore, repair, and replacement of damaged organ and tissue. Examples include terminally differentiated fibroblasts for cell therapy in skin repair, chondrocytes tested for osteoarthritis treatment, and hepatocytes from various sources for the creation of a bioartificial liver.

**FIGURE 4:**
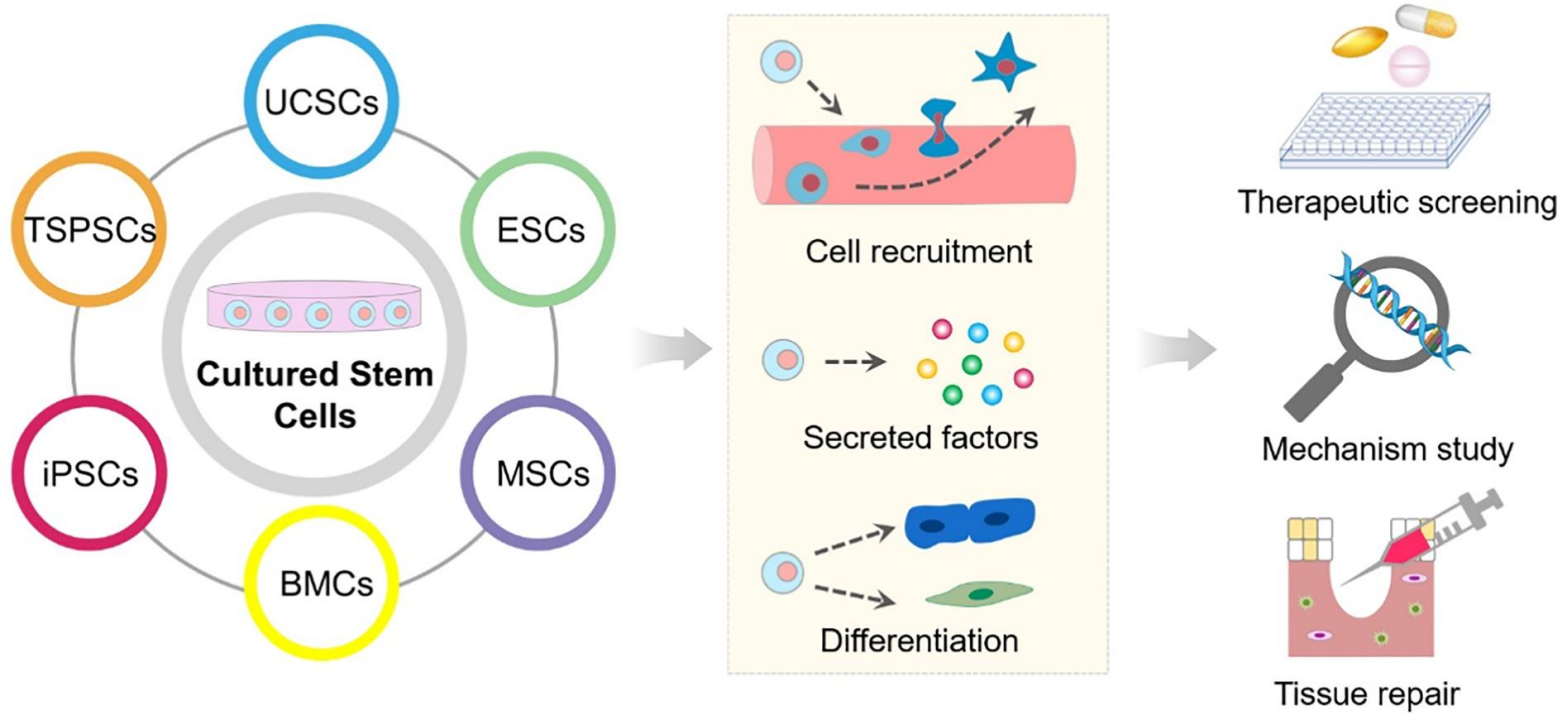
Biomedical applications of stem cells via cell recruitment, secreted factors, and cell differentiation. ESCs, embryonic stem cells; MSCs, mesenchymal stem cells; BMCs, bone marrow cells; iPSCs, induced pluripotent stem cells; TSPSCs, tissue specific progenitor stem cells; UCSCs, umbilical cord stem cells.

Compared to primary cells and genetically modified cells, stem cells as a highly promising cell source provide enormous opportunities for bioengineering (Figure 4). Stem cells have the capacity to self‐renew and differentiate to yield specialized cells in organs or tissues.^54^ Generally, stem cells could be classified into pluripotent stem cells (PSCs) and adult stem cells (ASCs) according to their source. ASCs are found in most adult tissues and are vital for tissue homeostasis, such as bone mesenchymal stem cells (MSCs) in bone marrow, neural progenitors in brain, and hair follicular stem cells in hair follicles. PSCs including embryonic stem cells (ESCs) and induced pluripotent stem cells (iPSCs) are capable of developing into the three primary germ cell layers of the early embryo, thereby potentially producing all cells of the human body.^55^ Until now, increasing evidence has demonstrated that cell and stem cell technology creates alternative avenues in regenerative medicine due to their extensive self‐renewal and pluripotent potential.

### 4.5 Organ transplant

Organ transplant technology aims to provide renewed life in individuals with damaged or missing organs.^56^ As one of the most successful advances in modern medicine, organ transplant has a relatively long history of operative skills. Up to now, various organs and tissues have been successfully transplanted worldwide, such as kidney, heart, skin, and cornea. Despite advances in medicine and technology, the unmet need for organ and tissue is far greater worldwide. To address this issue, bioengineered organs have been developed for human transplant. Specifically, human or animal organs (e.g., pig or cow) were decellularized while preserving the material's architecture, mechanical properties, and blood vessel networks.^57^, ^58^ Relevant cells from the organ's future recipient could be introduced into such a decellularized matrix to create functional organs, potentially minimizing organ rejection.

## 5 APPLICATIONS OF BIOMEDICAL ENGINEERING TECHNOLOGIES IN INFERTILITY

In the past several decades, biomedical engineers have participated in reproductive health from the mechanical studies of reproductive disorders to the design and development of diagnostic or treatment technologies. The following review will take a look at the development of engineering applied to reproductive health in a number of key areas. Each part is followed by examples of recent study and a brief discussion of new discoveries and expanded knowledge that will positively impact reproductive health in the near future.

### 5.1 Microfluidic‐based sperm analysis and selection

Sperm processing (such as cryopreservation, counting, and sperm‐sorting) for IVF is reported to have a negative impact on sperm attributes (semen volume, sperm morphology, sperm motility, viability, etc.).^59^ Thereby, many conventional sperm preparation techniques fail to obtain a sperm population with high motility and functions for ART. The centrifugation process involved has potential adverse effects on sperm quality and function because of mechanical stimulation and excessive levels of oxidative stress. In recent years, microfluidic techniques have been developed in this field to accurately estimate semen quality and predict its performance at insemination^59^, ^60^ (Table 1). Fertile Chip, as the new‐generation sperm selection method in intracytoplasmic sperm injection (ICSI) treatment, has been developed recently.^61^ The microchannels were designed to resemble the tubal channels, allowing for the selection of most progressive motile sperms. Also, clinical investigation has been conducted for couples with unexplained infertility and demonstrated no changes in fertilization rates, embryo quality, and pregnancy rates during IVF treatment in comparison to conventional swim‐up technique. Moreover, the study showed that the microfluidic‐based methods contributed to sperm selection with reduced DNA injury and fragmentation rates for ICSI.

**TABLE 1:** Microfluidic‐based sperm sorting and analysis for treatment of male infertility

Principle	Description	Sperm type	Analysis performed
Electrical impedance	Microdevice with an electrode gate	Mouse	Sperm concentration and differentiation between type of semen cell^62^
Oriented sperm swimming	Microdevice with dynamic flow	Human	Sperm motility and morphology^63^
Random swimming orientation	Microdevice with the optical system and analysis software	Pig	Sperm vitality and survival rate^64^
Resistive pulse technique	Microdevice with an induced electrical current and fluid flow through an electrode gate	Human	Sperm volume, sperm velocity, tail beat frequency, sperm head orientation, and shape^65^
Electrical impedance	Microdevice with electrode gates and dynamic flow	Pig	Presence of a defect, sperm direction, and sperm orientation^66^
Colorimetric signal	Paper‐based microdevice with a chemical‐based color scale	Human	Sperm concentration and motile sperm concentration^67^

**FIGURE 5:**
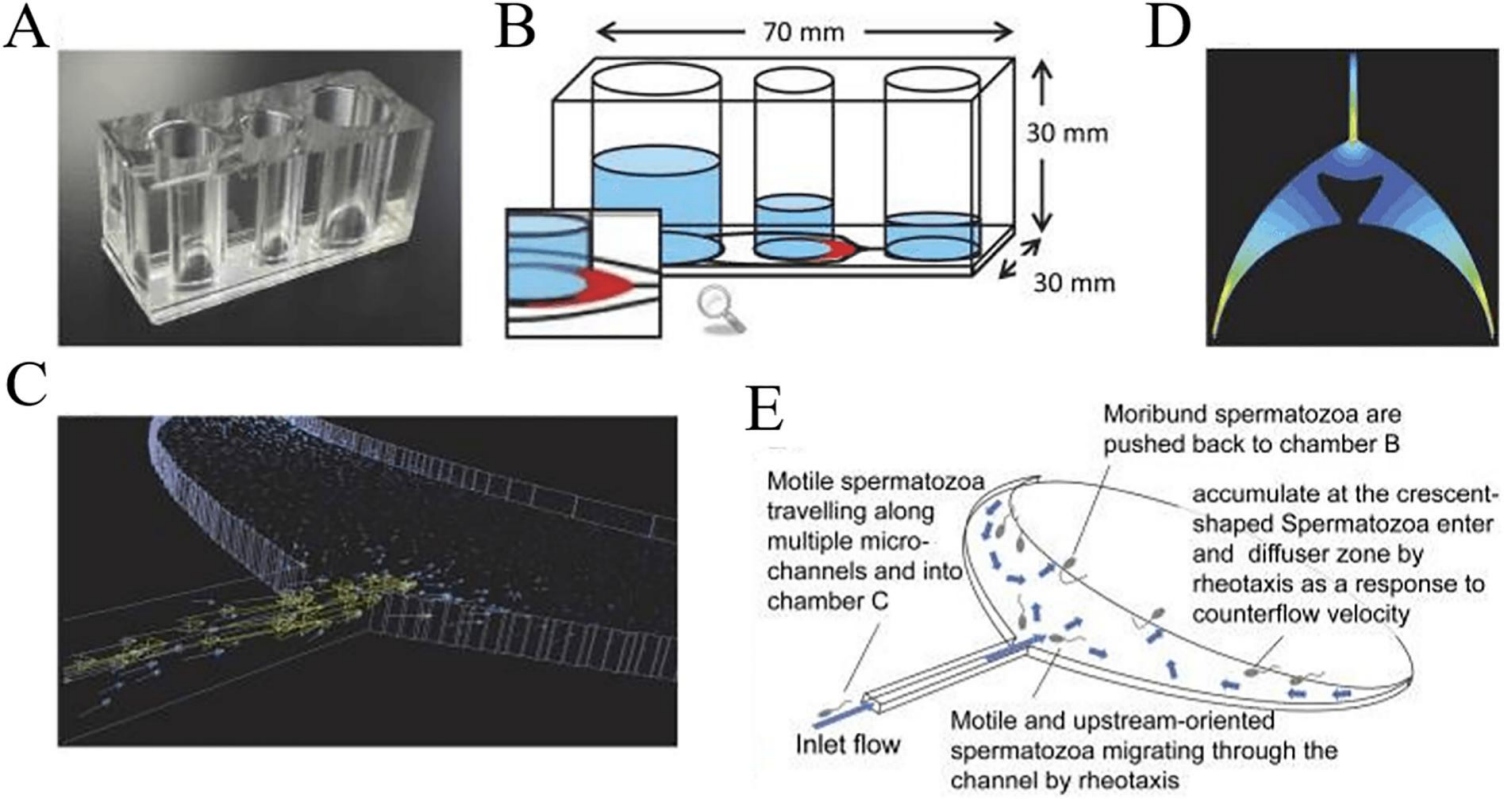
Schematic illustration of sperm sorting on a microfluidic chip with the crescent shape diffuser zone. (A) Photograph of the microfluidic chip microdevice. (B) Chambers and microchannels of the microdevice. (C) Simulation of the countercurrent flow. (D) The magnitude of the velocity in a diffuser zone. (E) Schematic for sperm sorting on the chip. Reproduced with permission.^68^  Copyright 2018, National Academy of Sciences.

Maria et al. reported a robust microfluidic sperm sorting system based on the precise control of fluid dynamics in space‐constricted environment^68^ (Figure 5A). Specifically, unidirectional and laminar or gradient flow can be created on chip, to some extent mimicking the variable fluidic environment in the female reproductive system (Figure 5B–D). As a barrier to the abnormal sperm, motile, DNA‐intact, and functionally competent sperms were isolated without potentially damaging forces such as centrifugation (Figure 5E). Considering a significant relationship between DNA fragmentation and fertility rate, we could foresee this technique helpful to solve the male infertility factor by sorting sperms with fertile attributes. In another experiment, a microfluidic sperm‐sorting chip was presented and utilized for sperm sorting.^69^ A total of 181 patients who underwent IVF because of male factor infertility were included in this study. The results show that the cost‐effective, disposable, and user‐friendly microfluidic device could improve IVF success rates, especially for male infertility.

### 5.2 Microfluidic‐based IVF

**FIGURE 6:**
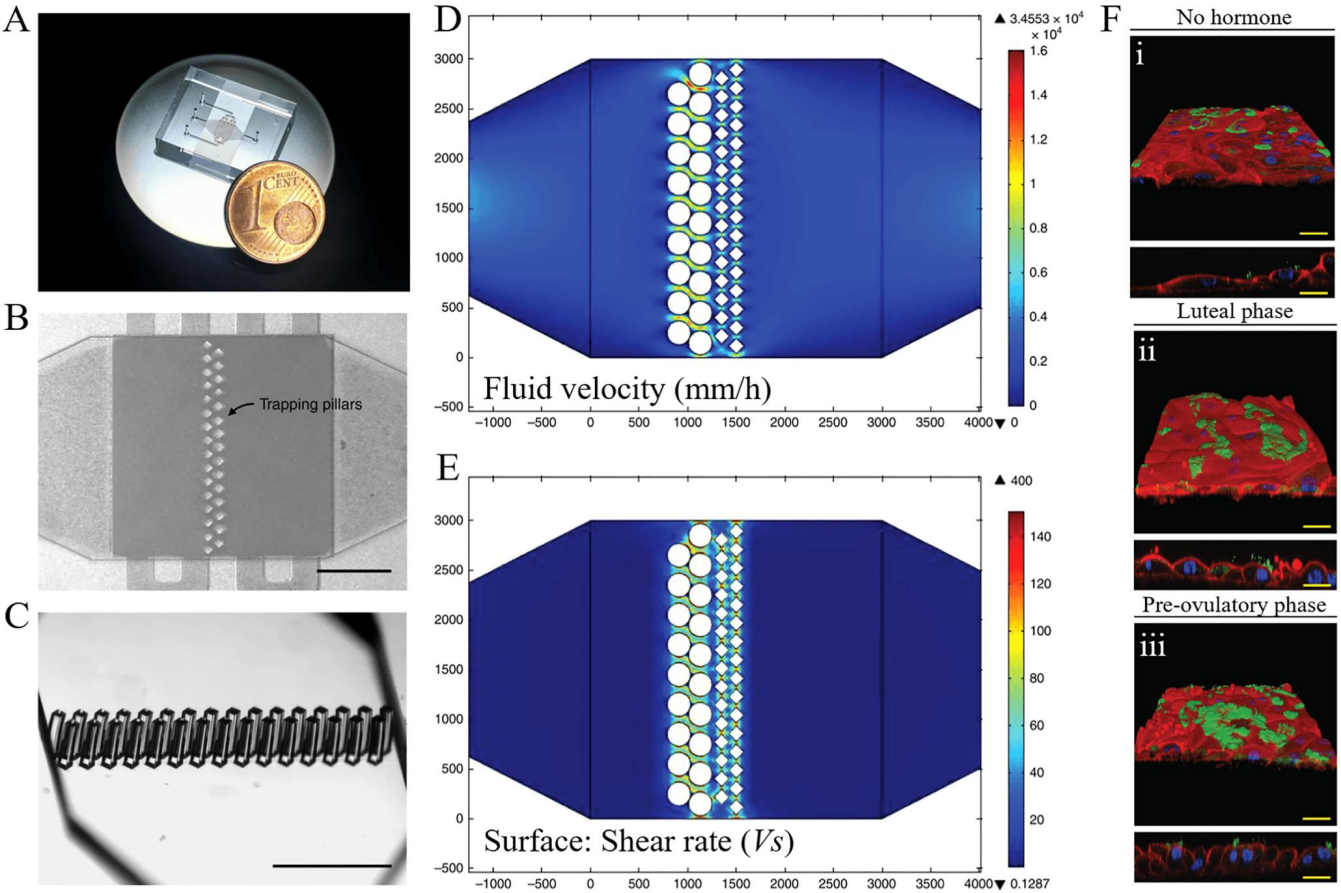
An oviduct‐on‐a‐chip with enhanced in vitro environment for zygote genome reprogramming. (A) Photograph of microfluidic chip microdevice. (B and C) The assembled microdevice with trapping pillars. (D and E) Simulation of the flow and shear rate on the chip. (F) Responses of 3D BOEC layer to hormone stimulation. Reproduced under terms of the CC‐BY license.^72^ Copyright 2018, The Authors, published by Springer Nature.

IVF is a process of fertilization where mature eggs retrieved from ovary are combined with sperms in a lab. The fertilized eggs are known as embryos and will be transferred to a uterus with the intention of establishing a successful pregnancy. Although a fairly new technology in the field of IVF, microfluidics has attracted considerable attention as a result of their potential applications. To date, there have been a significant amount of microfluidic fertilization devices that exceed some limits of conventional IVF techniques,^70^, ^71^ such as the stress imposed upon gametes and embryos, and high variability. In addition to numbers of embryos produced, time to pregnancy, and live birth rate, embryo quality is crucial to safeguard the health of IVF offspring. In this light, the oviduct‐on‐a‐chip microsystem has been developed, which created a near‐physiological microenvironment for fertilization and pre‐implantation development (Figure 6A–C).^72^ With dynamic flow, bovine oviduct epithelial cells (BOECs) exhibited villus‐like structures that recapitulated natural oviduct folding and could respond appropriately to the circulating hormone changes as observed in vivo (Figure 6D–F). Also, the oviduct‐on‐a‐chip supported fertilization and early embryo development until the 8–16 cells stage. The microfluidic device facilitated to produce more physiological zygotes than conventional in vitro zygotes in terms of their transcriptome and global DNA methylation pattern.

### 5.3 Engineering the microenvironment to improve in vitro oocyte maturation

**FIGURE 7:**
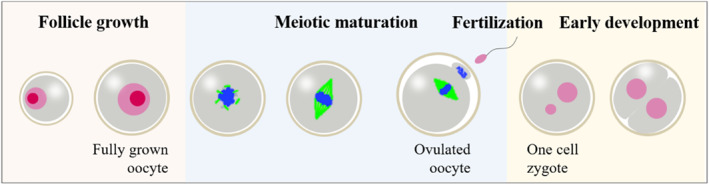
Schematic for oocyte maturation

Oocyte maturation is a critical step in the completion of female gametogenesis and thereby for successful fertilization and embryogenesis (Figure 7). Follicles are 3D spheres with a central oocyte surrounded by a granulosa cell layer, a basement matrix, and the outer theca cell layer. Multiple factors in the microenvironment have proven to be pivotal for oocyte maturation in humans and animals, including biomaterials, biochemical factors, and mechanical signals. To improve oocyte maturation and quality, engineers have developed novel techniques to create the proper microenvironment for ovarian follicle culture.^73^ Moreover, in vitro follicle culture systems could potentially advance our understanding of reproductive function and disease as well as improve clinical treatment.

#### 5.3.1 Biomaterials for follicle culture

**FIGURE 8:**
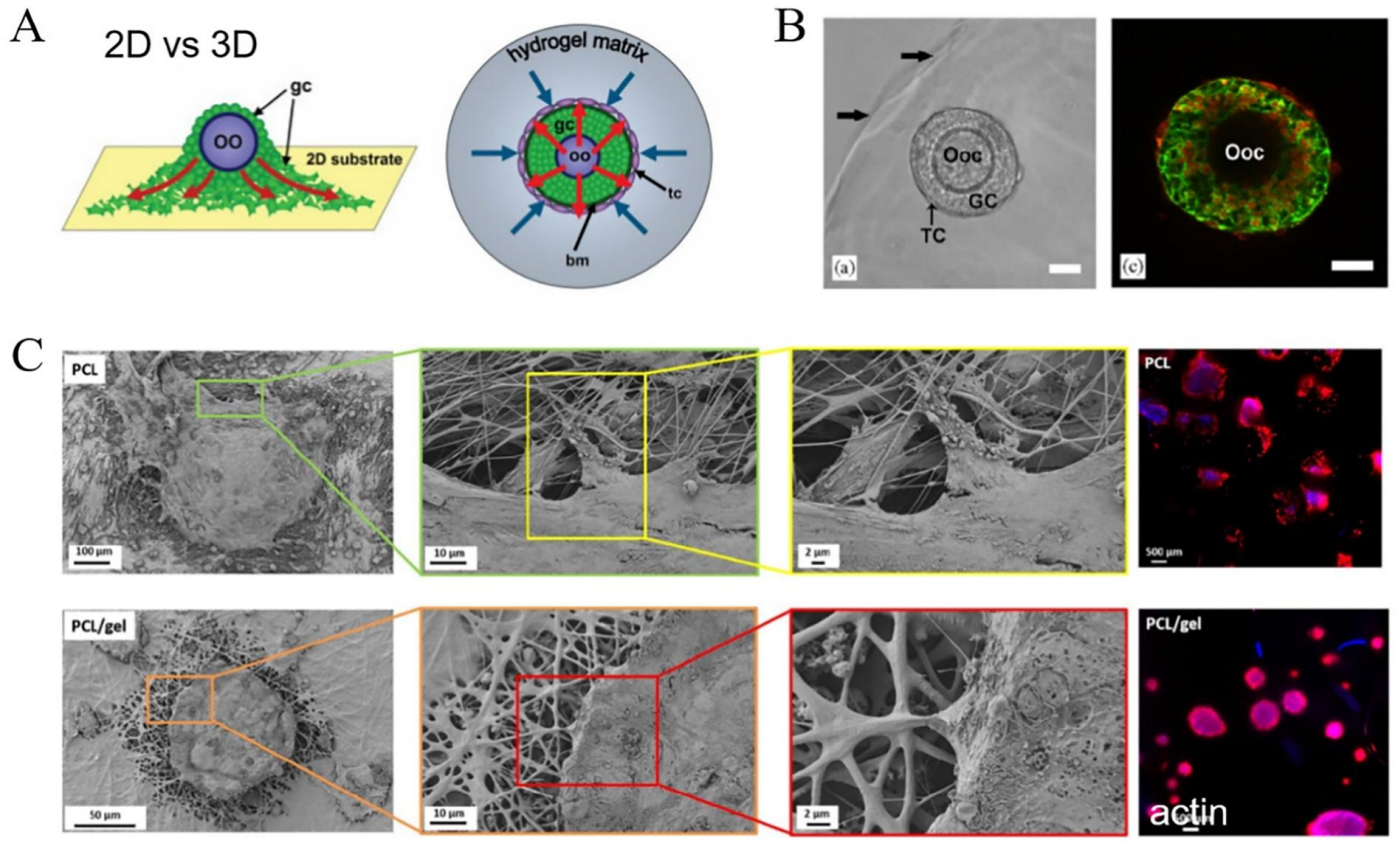
Engineering the follicle microenvironment. (A) Comparison of follicle architecture in 2D and 3D culture systems. Reproduced with permission.^75^ Copyright 2007, Georg Thieme Verlag KG. (B) Follicle encapsulated in an alginate bead. Reproduced with permission.^76^ Copyright 2006, Elsevier. (C) Follicle growth on an electrospun‐patterned porous scaffolds. oo, oocyte; Ooc, oocyte; GC, granulosa cell layers; TC, theca cell layer; PCL, poly(epsilon caprolactone).

Follicles are 3D spheres with multiple cell types in vivo; thus, the two‐dimensional (2D) culture system fails to support the production of embryos with high efficiency as the outer granulosa cells could attach to the culture dish and migrate away from the oocyte (Figure 8A). To date, many biomaterials have been utilized to create permissive 3D environment for follicle culture, including natural polymers, such as collagen, gelatin, fibrin, silk, and chitosan, and synthetic polymers, such as polyethylene glycol (PEG), poly (lactic acid), poly (glycolic acid), and poly (epsilon caprolactone) (PCL).^74^ The advantages of natural materials lie in their high bioactivity and biocompatibility, while synthetic materials are beneficial to design the exogenous ECM with well‐defined properties, such as mechanical and chemical signals, degradation, and anti‐inflammation activity. Alginate is a naturally occurring biopolymer by brown‐green algae and has been widely used in the follicle culture due to its gentle gelation and straightforward dissolution. Generally, follicles are encapsulated in alginate beads, which contribute to maintaining 3D their 3D structure relative to 2D culture systems^75^, ^76^ (Figure 8B). In vivo experiments have further proved that these follicles in alginate could promote oocyte fertilization and live births. Different from natural polymers, synthetic polymers are highly versatile in terms of morphological, mechanical, thermal, and degradation properties. Therefore, they are also widely applied to mimic the microenvironmental conditions. For instance, electrospun patterned porous PCL scaffolds have been developed to recapitulate the pore morphology and mechanical support, greatly contributing to the follicle growth and maturation^77^ (Figure 8C). Therefore, these elaborate biomaterials could promote follicle development and are highly potential for translation to clinical applications.

#### 5.3.2 Biochemical signals to direct follicle development

Ovarian folliculogenesis encompasses a wide breadth of extracellular signaling factors within the follicle microenvironment. The signals necessary for follicle development could be presented within the context of the biomaterials, including soluble and insoluble signals. Transport of these signals to the follicle is critical; thus, the biomaterial surrounding the follicle should allow for the efficient diffusion of nutrients and wastes.^78^ In order to address this issue, various questions must be considered, such as matrix type, the pores size of the matrix, concentration, and gelation treatments. Additionally, extracellular matrix (ECM) within the ovary is found to regulate the function of follicles by the cell–ECM interactions. However, follicle isolation disrupts these interactions in the outer layers of follicular cells. Recent and ongoing research is providing insight into diverse compositions and roles of matrix; thus, the proper biomaterials with defined ECM signals have been established. For instance, either intact proteins or small peptides have been coupled covalently to synthetic polymers, such as PEG and alginate, to convey bioactivity in the engineered scaffolds.^79^ The incorporation of these ECM signals was found to greatly facilitate the follicle growth and function as well as oocyte maturation.

#### 5.3.3 Matrix mechanical properties for oocyte maturation

A growing body of evidence indicates that mechanical stimuli are critical throughout the dynamic lifespan of the ovarian follicle by the bidirectional communication between cells and the surrounding microenvironment. In the case of the follicle, many in vitro research studies have demonstrated the complex roles of ovarian rigidity gradient in maintaining proper follicular architecture and growth. The well‐designed microenvironment aims to resemble the ovarian rigidity gradient and highlights the essential effects of ovarian biomechanics in oocyte maturation. Natural polymers have been extensively applied in tissue engineering, whereas the success of these materials for in vitro oocyte maturation has been modest. Thus, encapsulating matrices were subsequently developed by combining natural and synthetic polymers or modifying synthetic polymers. An interpenetrating network by fibrin and alginate has been developed with dynamic mechanical properties based on the gradual degradation of fibrin by cells, which meets the needs of the growing follicle.^80^ Also, with peptide cross‐linkers, modified PEG could be cross‐linked with a tunable degradation activity, allowing for the coordinated growth of multiple cells.^81^

### 5.4 Engineered device for embryo manipulation

#### 5.4.1 Microfluidic‐device for embryo culture

Over the past few decades, early embryo could be cultured in the laboratory with precise control of culture media, incubation/observation system, and oxygen level control. However, there is a growing body of evidence unveiled the important roles of biophysiological and chemical cues of the microenvironment in embryonic development. Accordingly, a series of the engineered culture systems have been developed, especially by combining microfluidic technology. Several microfluidic systems have demonstrated that a confined culture chamber with enhanced embryo density and less media greatly contributes to the embryonic development in comparison to conventional culture due to the enhanced growth factors.^82^ Additionally, microfluidic could create an in vivo‐like dynamic fluid environment, which displayed a greater number of blastocysts with a lower percentage of degenerated embryos in comparison to those with controlled microdrops.^83^ While the benefits of engineered technologies in embryo culture have been shown, extended work still needs to be performed with more convincing results for clinical and commercial use.

#### 5.4.2 3D printing for embryo injection and screening

Many studies have reported the application of 3D printing in microfluidic device fabrication for cell culture, drug screening, and clinical analysis. In clinic, developmental biology requires rapid manipulation, including embryo injection and screening. Thus, a series of 3D printable tools have been developed in recent years and some have been commercially available. A device with two stamps was designed and prepared for the increased injection and screening of embryos,^84^ potentially acting as a feasible platform for clinical applications. Despite the progress, some biocompatible 3D printing machines were proven to cause developmental toxicity in embryo culture, which was evidenced by *Danio rerio* embryo culture.^85^ These findings demonstrate that there is still a long way to go before the extensive use of 3D printed device in developmental biology (Table 2).

**TABLE 2:** 3D printing‐based embryo manipulation

Materials	Application	Instrumentation	Species	Parameters
Photosensitive resin	Embryo arraying^86^	Vacuum pump	Zebrafish	Plating time, cost, effect on the embryos, and required amount of liquid
Agarose	Embryo injection and screening^84^	n/s	*Crepidula fonicata*, *Parasteatoda tepidariorum*, *Xenopus laevis*, and *Danio rerio*	Injection and screening speed, effect on the embryos
PEGDA	HET CAM assay^87^	Micropump, camera	Chicken	Biocompatibility and cytocompatibility
Photosensitive resin	Embryo culture^85^	n/s	*Danio rerio*	Cytotoxicity

Abbreviations: HET CAM assay, hen’s egg test chorioallantoic membrane; n/s, not specified.

### 5.5 Bioscaffolds for reproductive tissue repair

#### 5.5.1 Male reproductive tissue repair

**FIGURE 9:**
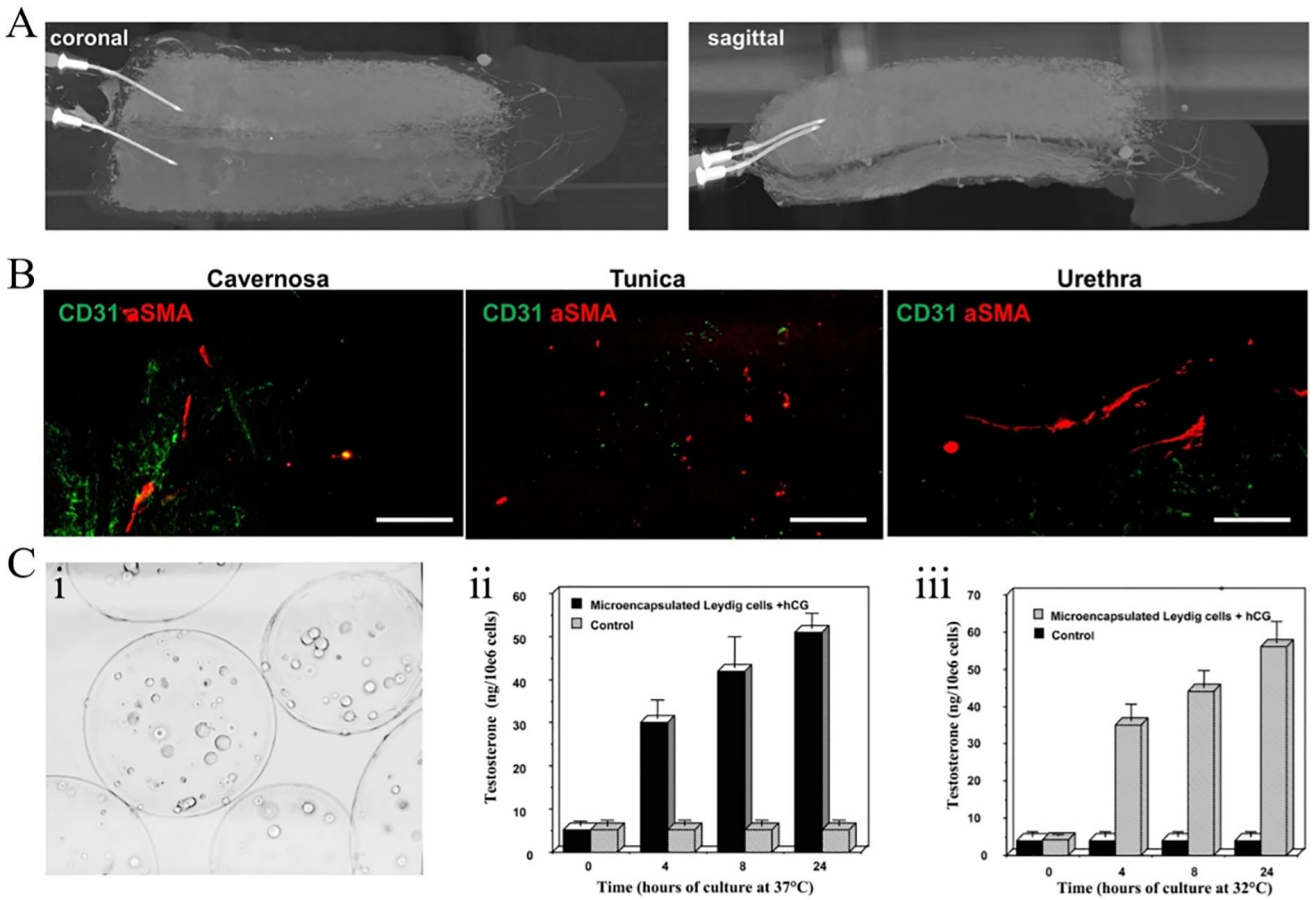
Bioscaffolds for male reproductive disorders. (A and B) Complete human penile scaffold for tissue engineering. Reproduced under terms of the CC‐BY license.^90^ Copyright 2019, The Authors, published by Springer Nature. (C) Microencapsulation of Leydig cells in microspheres for testosterone supplementation. Reproduced with permission.^91^ Copyright 2003, Oxford University Press.

Organ‐preserving surgery is a reasonable approach in managing a wide variety of penile disorders, such as penile cancer, trauma, and congenital anomalies.^88^ Therefore, phallic reconstruction is a possible option to create a penis that enables resumption of sexual activity,^89^ but the major limitation is the availability of the radial forearm and graft material with a low risk of prosthesis erosion or infection. Currently, autologous tissue, derived from the patient's own cells, has been employed for phallic reconstruction. Specifically, acellular corporal matrices were prepared from donor rabbit penis and rich of collagen, which facilitated the adhesion and growth of tissue‐specific cells, including corpus cavernosal smooth muscle cells and endothelial cells.^90^ After implantation for 6 weeks, the engineered corpora cavernosa demonstrated adequate structural and functional parameters. Of particular note is that the animals with the engineered corpora resumed the normal mating activity by 1 month after implantation, as sperms were observed in animals with the engineered corpora. Notably, these mated female rabbits finally conceive and delivered healthy pups, showing the great potential of engineered autologous tissues in animals. Consider the anatomical and functional complexity of human penile, phallic reconstruction presents unique challenges in humans. Tan et al. have established the first protocol for decellularizing the complete human phallus (Figure 9A,B), which represents a novel solution to total penile loss.^90^ These research studies have proven the feasibility of the engineered penile corpora cavernosa tissue.

Testicular dysfunction may occur when the testicles produce low male sex hormone testosterone or sperm. Testosterone replacement therapy (TRT) is required for the treatment of some forms of testicular dysfunction, which could be given as a gel, patch, injection, or implant.^92^ Of note is that long‐term nonpulsatile TRT is not optimal, and clinical complications, including excessive erythropoiesis and bone density changes, are not negligible. To overcome this issue, an engineered system was established by microencapsulating testosterone‐secretive Leydig cells in alginate microcapsules (Figure 9C).^91^ After being injected into castrated animals, testosterone levels could keep stable for a long time. In clinic, patients require persistent TRT after receiving testicular prosthesis implantation. Currently, many studies have created testicular prostheses with ability to release testosterone into the bloodstream in a controlled manner after implantation. Silastic‐based materials are proved to be ideal for testicular prostheses as well as hormone‐releasing carriers and have increasingly become a popular topic for the treatment of testicular dysfunction.

#### 5.5.2 Uterine tissue repair

Bioscaffolds have been developed to reconstruct the injured uterus through the combination of cells, stem cells, chemicals, and biomaterials. Y. Zhu et al. have designed a novel gelatin methacryloyl (GelMA) microneedle (MN) patch loaded with antioxidant nanozyme and stem cells.^93^ Of note, nanozyme is an emerging nanomaterial featured with intrinsic enzyme‐like activities. Herein, cerium oxide (CeO_2_) was utilized, which had excellent functions to remove ROS, potentially inhibiting the inflammatory responses at the injured sites. When implanted, the multifunctional MN patch was observed to significantly enhance endometrial repair and exhibit the increased pregnancy rates in Asherman's syndrome (AS) model rats. These results proved that this novel MN patch enhanced morphological and functional reconstruction of the injured endometrium.

**FIGURE 10:**
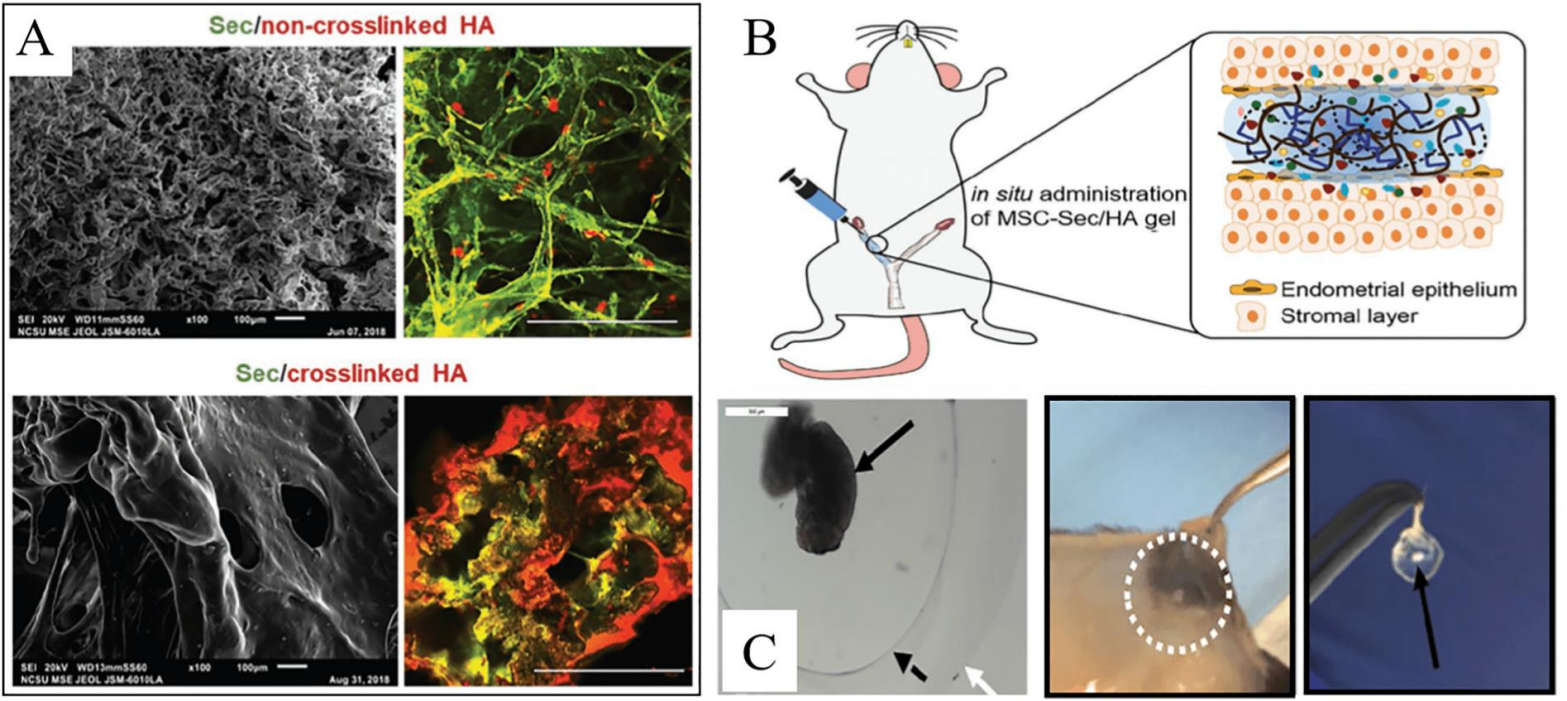
(A and B) HA hydrogel loaded with MSC‐secretome for endometrial repair in the AS rat model. Reproduced with permission.^95^ Copyright 2019, John Wiley and Sons. (C) Ovarian encapsulation in degradable PEG capsules with enhanced endocrine function and inhibited immune rejection. Reproduced under terms of the CC‐BY license.^98^ Copyright 2019, The Authors, published by Springer Nature.

In addition to biocompatible materials and cells, several factors should also be considered, such as vascularization, hormones, and signaling molecules, due to the complex structural and mechanical signals in vivo. Li et al. presented a collagen scaffold loaded with human endometrial perivascular cells overexpressing CYR61, a protein with an important role in vascular development. As expected, the engineered stem cell system enhanced endometrial and myometrial regeneration and promoted neovascular regeneration in injured rat uteri. Lei et al. developed an angiogenic hydrogel microsphere for the treatment of thin endometrium.^94^ As the carrier for VEGF, methacrylated hyaluronic acid (HAMA) microspheres were found to enhance angiogenesis at the injured sites and have excellent therapeutic effects in the mouse model. Besides, recent biological studies have revealed that conditioned media with stem cells might be a safe and promising alternative for cell therapy. Some researchers have applied MSC secretome (MSC‐Sec) for the treatment of injured endometrium and the prevention of AS (Figure 10A‐B),^95^ potentially overcoming some limits of conventional stem cell therapy, such as tumor formation, low efficiency of cell targeting to the injured site. Liu et al. proposed a novel strategy by loading crosslinked hyaluronic acid gel with MSC‐Sec.^95^ The engineered system allows for the sustained release of secretome and facilitates the uterine repair with enhanced successful pregnancy rates based on the in vivo studies.

#### 5.5.3 Ovary tissue repair

Ovary is a heterogeneous organ that compartmentalizes different follicle pools (from primary to mature follicle) into the cortex and medulla regions with varied mechanical properties. Considering the size of human ovarian transplants, 3D printing could act as a versatile tool to address all of these implant requirements by creating a mimetic construct with well‐defined mechanical features. Laronda et al. have 3D printed a highly promising bioprosthetic ovary using microporous hydrogel scaffolds.^96^ Most importantly, in vivo studies signified that the bioprosthetic ovary was highly vascularized and active in the reproductive axis. When implanted, the ovarian function was fully restored, including ovulation of healthy eggs, hormone secretion, as well as high rate of live birth. For POI therapy, autotransplantation of cryopreserved ovarian tissue and HRT is available at present, and thus far, engineered technologies have been incorporated. Kim et al. have engineered an artificial ovarian tissue from preantral follicles using a synthetic tunable hydrogel, PEG vinyl sulfone (PEG‐VS), as a supportive matrix, which has been found to promote ovarian functions in vivo.^97^ Additionally, the novel immune‐isolation hydrogels were designed and fabricated, which could restore ovarian endocrine functions and preclude immune rejection. May et al. constructed a dual microcapsule with a proteolytically degradable core, which was conductive for the dynamic growth of ovarian tissue (Figure 10C).^98^ The nondegradable PEG shell could serve as an important physical barrier, promoting graft survival and ovarian endocrine restoration. This biomaterial system might provide a promising platform for female cancer survivors with POI. These patients could receive donor ovarian tissue, which could either induce puberty or restore physiological hormonal balance, potentially addressing limitations to current therapy.

#### 5.5.4 Cervicovaginal tissue repair

Cervical agenesis or cervical dysgenesis is implicated in a significant number of infertility and preterm birth. Once detected, cervical cerclage is the typical treatment, but current studies on its efficiency are controversial with conclusions drawn from clinical evidence as opposed to mechanical properties. To better understand the mechanical limitations, synthetic silicone rubber cervices were fabricated and utilized to investigate the effects of material mechanics on the integrity of the cerclage.^99^ Recently, cervical reconstruction using biomaterial grafts has been applied successfully in congential agenesis of the cervix. A patient was diagnosed with cervical hypoplasia and dysmenorrhea, and cervical reconstruction was necessary to preserve her reproductive potential. Thus, a polytetrafluoroethylene (PTFE) ring graft was designed and lined with vaginal mucosa to stent open the neocervical canal. Notably, these patients with outcomes up to 6‐month clinical follow‐up have resumed normal activities, including mating and menses.^100^ In addition, split thickness skin graft and pudendal thigh flaps have been reported for cervicovaginal reconstruction with good success.^101^

### 5.6 Cell‐based approaches to restore reproductive tissue function

Endogenous tissue repair takes place involving a series of coordinated events, and interruption or deregulation of any events may lead wounds to become chronic and abnormal scar formation. Recent investigations have exhibited that cells and stem cells could stimulate the repair and regeneration of injured tissues or organs, motivating a growing number of cell‐based interventions in tissue regeneration.^102^ Engineering cell therapeutic therapies for skin, musculoskeletal tissue repair have been reviewed in other studies.^103^, ^104^ Here, we then present current clinical achievements where cells and stem cells have been applied to restore reproductive tissue function.

#### 5.6.1 Enhanced male reproductive organ function

Nowadays, MSC therapy offers a broad spectrum of treatment for male subfertility and infertility.^105^ Testicular stem cells serve as a reserve storage, which could divide asymmetrically and give rise to progenitor cells. The transplanted MSCs could interact with these cells and facilitate fertility restoration in vivo. For example, nonobstructive azoospermia (NOA) is a reproductive disease in men that affects about 10% infertile men. In vitro studies have demonstrated that NOA can be reversed based on MSC differentiation into male germ cells by the integration with growth factors, chemical components, cell co‐culture, and genetic manipulation.^106^ NOA animal models even exhibited the efficient induction of spermatogenesis and/or differentiation of MSCs into germ cells in the testes. Testicular function restoration by MSC transplantation might attribute to the reduced apoptosis and oxidative stress, the differentiation into target cells, as well as the secretion of growth factors. Based on these studies, various clinical trials for treating azoospermia by MSC transplantation has been recorded. Jordan scientists have demonstrated the therapeutic effects of intratesticular injections of CD34/CD133 MSCs in azoospermia men. Also, several clinical studies have been recruited for infertility treatment by MSC therapy in the United States and Iran.

#### 5.6.2 Enhanced uterine function

**FIGURE 11:**
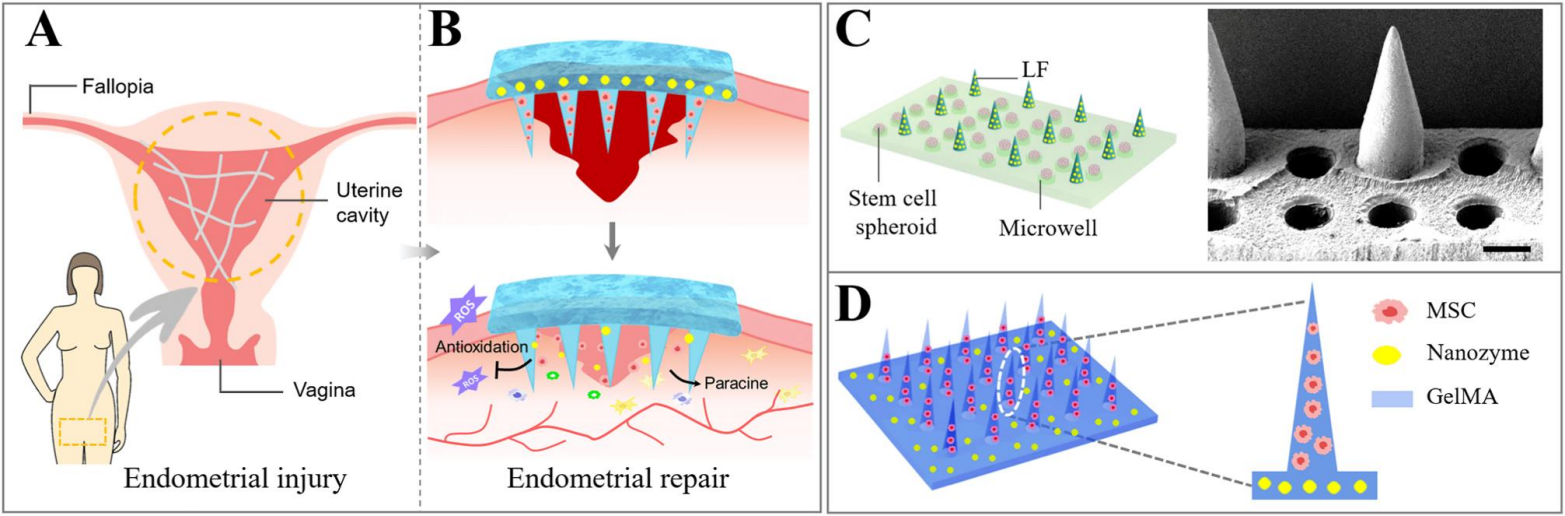
(A and B) Schematic for microneedle‐based endometrial repair. Reproduced with permission.^93^ Copyright 2022, Elsevier. (C and D) Design of multifunctional microneedle patch loaded with MSC. Reproduced with permission.^110^ Copyright 2022, John Wiley and Sons.

The endometrium is a dynamic tissue with multipotent stem cells to regenerate the endometrial stroma during each menstrual cycle. Thus far, stem cell transplantation has been evolving to improve current surgical techniques for uterine repair. Many researchers have focused on MSCs therapy to restore the uterine function and reverse the damages caused by AS. Increasing evidence have indicated that the paracrine signaling molecules secreted by MSCs imparted their therapeutic effects in the injured uterine. A series of studies have displayed the increased endometrial thickness and vascularity following the stem cell injections. Human amniotic MSCs,^107^ umbilical cord MSCs, chorionic villi MSC,^108^ and menstrual blood‐derived stromal cells ^109^ have proven to be effective in the treatment of intrauterine adhesions. Moreover, novel multifunctional MN patches were designed and prepared by Zhu and Li et al., which were found to enhance endometrial repair in rats (Figure 11).^93^, ^110^ In comparison to dissociated cells, MSC cell spheroids had significant advantages of cell proliferation, cell differentiation, and cell migration. This study exhibited the extensive application prospects of cells and biomaterials in tissue repair. In clinic, combined with hormonal stimulation, the endometria of all seven patients showed significant proliferation. Three patients eventually became pregnant and delivered live babies successfully. In addition to the enhanced uterine function, studies also show that the stem cell could promote endometriosis due to the intrinsic characteristics, including high proliferation, self‐renewing, and high plasticity.

#### 5.6.3 Enhanced ovary function

**FIGURE 12:**
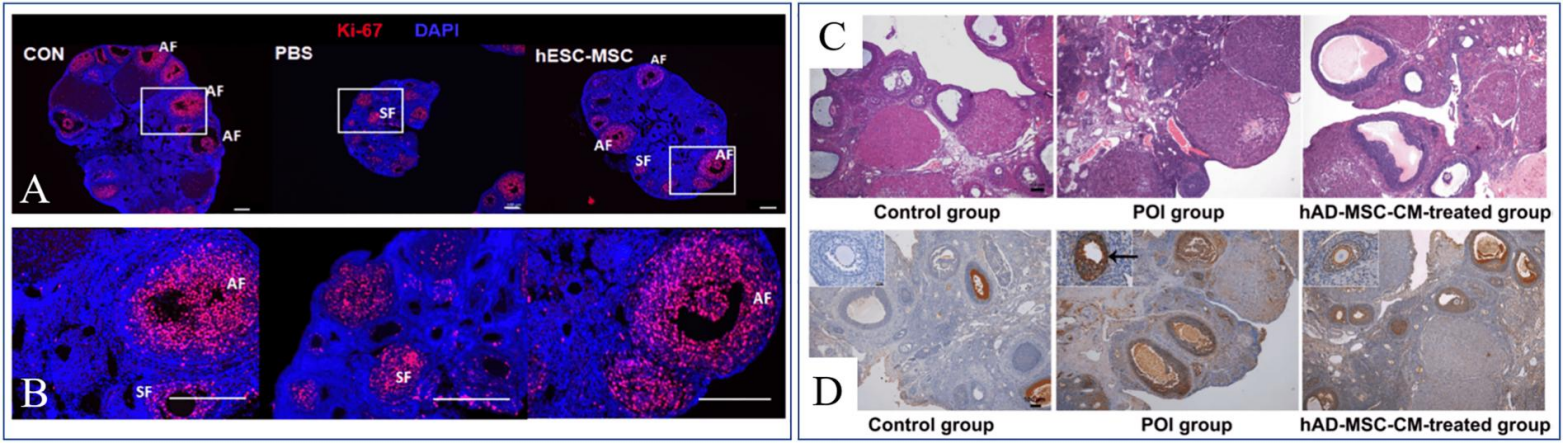
(A and B) hESC‐MSCs promoted recovery of ovarian functions in POI model mice. Reproduced under terms of the CC‐BY license.^112^ Copyright 2020, The Authors, published by Springer Nature. (C and D) hAD‐MSCs improves the ovarian function in POI model rats by paracrine. Reproduced under terms of the CC‐BY license.^113^ Copyright 2019, The Authors, published by Springer Nature.

MSC‐based clinical therapy as a potential alternative treatment modality has recently been applied to restore damaged ovarian functions.^111^ Several key features of the enhanced ovary functions have been measured, such as folliculogenesis, granulosa cell apoptosis, vascular formation, the pregnancy rate, and hormone levels. Many stem cell‐based therapies attenuate the effects of aging on reproductive health, restores the capacity for embryo development, and reestablishes the regulation of inflammation and apoptosis. It is reported that hESCs‐derived MSCs (hESC‐MSCs) contributed to restore the structure and function in cisplatin‐induced POI mice (Figure 12A,B).^112^ Also, human amnion‐derived MSCs (hAD‐MSCs) were identified to improve the ovarian function in chemotherapy‐induced POI rats driven by paracrine mechanisms (Figure 12C,D).^113^ Considering the immense promise of cell therapy in the treatment of ovarian dysfunction, the investigators in many countries, such as China (https://clinicaltrials.gov/ct2/show/NCT03877471), have conducted clinical trials to evaluate the efficiency and safety of MSC‐based cell therapy in women suffering from POI. Much more research and clinical results are needed to understand the full nature and potential of stem cell therapies as future medical therapies for POI.

#### 5.6.4 Enhanced cervicovaginal function

Pelvic organ prolapse (POP) refers to the drop of one or more organs in the pelvis slip (the vagina, cervix, uterus, bladder, and rectum), which accounts for 10%–30% women, especially in postmenopausal women.^114^ Although POP is a nonfatal disease, the routine surgical therapy is aimed to only provide support for the vaginal tissue and often accompanied with the high risk of recurrence. In recent years, stem cell‐based regenerative therapy might provide a novel therapeutic strategy for POP. Zhang et al. injected MSCs from human umbilical cord into bilaterally ovariectomized rhesus macaques.^115^ Significantly higher collagen composition, elastic fiber, smooth muscle, and microvascular density were observed in the MSC group, suggestive of the great potential of MSCs in the reconstruction and restoration of injured vaginal tissue. Mayer‐Rokitansky‐Küster‐Hauser syndrome (MRKH) is a rare disorder found in females, characterized by congenital aplasia of the upper vagina and uterus. McIndoe vaginoplasty is widely performed to treat MRKH by creating a vaginal canal covered with a full‐thickness skin graft.^116^ Also, amniotic membranes,^117^ inert materials,^118^ and oral mucosa^119^ have been utilized for vaginoplasty in some pilot studies. Marchese et al. presented the autologous in vitro culture vaginal tissue, and human vaginal mucosa cells (HVMs) from the tissues were highly proliferative and exhibited key epithelial features, allowing for the efficient epithelialization of the neovagina walls. Thus, this modified cell‐based therapy could provide a simple and effective manner to create a neovagina with near‐physiological characteristics.^120^

## 6 CONCLUSION AND PERSPECTIVE

In this review, we have presented a comprehensive look at recent advances in reproductive medicine based on biomedical engineering technologies. Due to the considerable advantages, biomedical engineering technologies have demonstrated significant potential in the treatment of male and female infertility. These technologies could be classified into microfluidic technology, organ transplant, biomaterials, cell and stem cell technology, and 3D printing and have been widely applied for fundamental studies and clinical therapies in recent years. We discussed the applications of these technologies in diagnostics and treatment of male and female infertility and emphasized the efforts to solve troubles raised in any of the steps necessary for a successful pregnancy. Comparing with conventional methods, bioengineered systems are advantageous in sperm analysis, in vitro fertilization, in vitro oocyte maturation, embryo culture, and reproductive tissue repair with high flexibility, efficiency, safety, and low cost. Also, the efforts in translational research from laboratory to clinical practice were highlighted.

In spite of many investigations into biomedical engineered therapies, the feasibility for clinical treatment still needs to be demonstrated. Firstly, more basic research is required for the full understanding of the biological mechanisms for successful pregnancy and related disorders. It is well‐known that human pregnancy is highly complex involved with paternal, maternal, and fetal systems, and many troubles could arise in any of the steps possibly cause subfertility and infertility. Therefore, the cellular and molecular interactions in physiological and pathological conditions should be investigated in depth. Although a broad spectrum of basic studies has been conducted in the past several decades, there is still space for a full evaluation of these complicated and dynamic interactions. Accordingly, elaborate designs of the engineered system for clinical treatment are largely limited, thereby affecting the subsequent biomedical applications. Secondly, in many respects, research findings are only as valuable as how well they can be put into practice to improve outcomes. Undoubtedly, bioengineering technology acts as a promising platform to put rather complex findings into practice. As an interdisciplinary field, bioengineering technology combines basic and applied sciences. To construct a bioengineered system in areas of reproductive medicine, a series of elements should be considered, such as biomaterials, biocompatibility, degradation, cost, morphology, mechanics, and components. Notably, due to the complex structure and the specialized function of reproductive organs, especially the ovary and testis, reliable techniques should be developed to enhance both the morphological and functional reconstruction. From this point of view, there are still numerous obstacles in terms of biocompatibility and safety. Last but not least, it should be pointed out that the ultimate goal of bioengineering technology is to demonstrate its value in clinical practice. Nevertheless, clinical studies have been seldomly conducted up to now due to potential risks and ethical issues. Alongside patients' increased participation in research, attention is being drawn to ethical issues in different research contexts. In many countries, including China, the approval of research practice must be obtained from the institutional or professional procedural ethics review committee, ensuring research is ethical throughout the duration. Clinical research often confronts challenges and dilemmas arising when the study is underway and hence could not have been anticipated, or guarded against, at the outset.

Overall, bioengineering strategies display enormous promise as potential treatments and cures for male and female infertility. We believe that the above‐mentioned challenges of bioengineering technologies could motivate the development of basic and applied science, and great contributions could be made to push forward the clinical translation of these technologies. We expect that the development of bioengineering technologies could be applied in more areas and benefit the medical community in the foreseeable future.

## AUTHOR CONTRIBUTIONS

Yuanjin Zhao conceived the study. Yujuan Zhu wrote the manuscript. Bin Kong and Rui Liu helped to revise the manuscript. All authors read and approved the final manuscript.

## CONFLICT OF INTEREST

The authors declare that they have no known competing financial interests or personal relationships that could have appeared to influence the work reported in this paper. Yuanjin Zhao is a member of the *Smart Medicine* editorial board.
